# Macro-level efficiency of health expenditure: Estimates for 15 major economies

**DOI:** 10.1016/j.socscimed.2021.114270

**Published:** 2021-10

**Authors:** Simiao Chen, Michael Kuhn, Klaus Prettner, David E. Bloom, Chen Wang

**Affiliations:** a Chinese Academy of Medical Sciences & Peking Union Medical College, Beijing, China; b Heidelberg Institute of Global Health, Faculty of Medicine and University Hospital, Heidelberg University, Heidelberg, Germany; c International Institute for Applied Systems Analysis (IIASA), Laxenburg, Austria; d Wittgenstein Centre (IIASA, OeAW, University of Vienna), Vienna Institute of Demography, Vienna, Austria; e Vienna University of Economics and Business (WU), Department of Economics, Vienna, Austria; f Department of Global Health and Population, Harvard T.H. Chan School of Public Health, Boston, MA, USA; g National Clinical Research Center for Respiratory Diseases, Beijing, China; h Department of Pulmonary and Critical Care Medicine, Center of Respiratory Medicine, China–Japan Friendship Hospital, Beijing, China; i Chinese Academy of Engineering, Beijing, China

**Keywords:** Health expenditure, Efficiency, Value of statistical life, Welfare, Macroeconomics, Health system, Macro-efficiency test

## Abstract

The coronavirus disease 2019 (COVID-19) pandemic highlights the importance of strong and resilient health systems. Yet how much a society should spend on healthcare is difficult to determine because additional health expenditures imply lower expenditures on other types of consumption. Furthermore, the welfare-maximizing (“efficient”) aggregate amount and composition of health expenditures depend on efficiency concepts at three levels that often get blurred in the debate. While the understanding of efficiency is good at the micro- and meso-levels—that is, relating to minimal spending for a given bundle of treatments and to the optimal mix of different treatments, respectively—this understanding rarely links to the efficiency of aggregate health expenditure at the macroeconomic level. While micro- and meso-efficiency are necessary for macro-efficiency, they are not sufficient. We propose a novel framework of a macro-efficiency score to assess welfare-maximizing aggregate health expenditure. This allows us to assess the extent to which selected major economies underspend or overspend on health relative to their gross domestic products per capita. We find that all economies under consideration underspend on healthcare with the exception of the United States. Underspending is particularly severe in China, India, and the Russian Federation. Our study emphasizes that the major and urgent issue in many countries is underspending on health at the macroeconomic level, rather than containing costs at the microeconomic level.

## Highlights

• Macroeconomic efficiency of health expenditure is rarely explored.
• We propose a framework to assess the macro-level efficiency of health expenditure.
• We find that underinvestment in health at the macroeconomic level is a major issue.
• Underspending is particularly severe in China, India, and the Russian Federation.
• The US is the only country exhibiting overspending.

## 1 Introduction

To tackle health emergencies, such as the coronavirus disease 2019 (COVID-19) pandemic, and to cope with the health-related challenges of the unprecedented population aging that the world currently faces (Bloom et al., 2015, 2018, 2020; Chen and Bloom, 2019; Chen et al., 2018, 2019b), nations need strong and resilient health systems. Countries’ health expenditures increased strongly in recent decades, not just in absolute terms but also as shares of their gross domestic products (GDPs) (Papanicolas et al., 2018). This increase in global health expenditure is expected to continue over the coming years and to reach more than US$24 trillion by 2040 (Dieleman et al., 2017). Yet the important question of whether such high spending levels are optimal remains largely unanswered. While one analysis suggests that the spending level for the United States may be too low (Hall and Jones, 2007), this result remains up for debate (Cutler, 2021) and systematic and rigorous investigations of other countries are scarce.

Facing growing health expenditures, many researchers question the efficiency of the healthcare sector. Previous studies suggest that inefficiencies exist in the production, organization, and administration of healthcare; in the allocation of health expenditure across services and sub-groups of the population; and in the adoption of new technologies (Baicker et al., 2012; Berwick and Hackbarth, 2012; Chandra and Skinner, 2012; Chandra and Staiger, 2020; Cutler, 2018; Cutler and Ly, 2011; Garber and Skinner, 2008). However, these studies mainly focus on micro-level (or production) efficiency, i.e. the production of a given volume and type of healthcare using minimal inputs, or on meso-level (or allocative) efficiency in the sense of a welfare-maximizing mix of healthcare services for a given aggregate healthcare budget (Jacobs et al., 2006; World Health Organization, 2016). Few studies have explored the macro-level efficiency of health expenditure in the sense of a welfare-maximizing mix of aggregate health expenditure and spending on other goods and services (consumption in particular).

Exploring macro-level efficiency of health expenditure is crucial for policymaking. Decisions based solely on the micro-level and meso-level efficiency of health expenditure may lead to the wide implementation of cost-containment strategies such as regulating drug prices, lowering costs in public hospitals, and setting spending caps for healthcare services (Busse and Blümel, 2014; Busse et al., 2017; Busse et al., 2013; Garber et al., 2007; Li, 2011; Liu et al., 2017; Liu et al., 2019; Mossialos and Le Grand, 2019; Stabile et al., 2013; Stadhouders et al., 2019; Yip et al., 2019) that could be counterproductive from an aggregate perspective. This is because, at the aggregate level, the share of health expenditure in GDP may be inefficiently low and cost-containment strategies may exacerbate this problem, inter alia, by impeding quality and innovation in healthcare. To understand why, it is important to recognize that two fundamentally different factors drive high and growing health expenditure shares. These factors are (i) overspending due to inefficiency in the production and allocation of healthcare and (ii) a high demand for efficiently provided healthcare that grows even further in times of population aging. Separating efficient from inefficient spending increases is challenging both conceptually and practically.

In this article we focus on the macroeconomic efficiency of health expenditure, i.e., whether countries underspend or overspend on healthcare relative to all other types of consumption. We provide a novel and simple way to test for macro-efficiency and determine the extent to which the following important large economies—Argentina, Australia, Brazil, Canada, China, France, Germany, India, Italy, Japan, the Russian Federation, South Africa, Spain, the United Kingdom, and the United States—underspend or overspend on health at the macro level.

## 2 Methodology

### 2.1 Data sources

To perform the test for 15 large economies, we rely on World Development Indicators (2021) data on life expectancy, consumption expenditures as a share of GDP, health expenditures as a share of GDP, and GDP itself (World Bank, 2021). In the main analysis, we use data for 2015 and, in further analysis, we also use 2010 data to show how the macro-efficiency score changed over time. As a specification for instantaneous utility, we apply a standard isoelastic utility function. As the inverse of the elasticity of intertemporal substitution, i.e., a measure of how willing households are to sacrifice consumption today in exchange for consumption tomorrow, we use the value 1.01, which is well in line with empirical evidence (Chetty, 2006). In addition, we consider that individuals discount their future utility at a rate of 2.5 % (Kuhn and Prettner, 2016). The main text provides details of our estimation of the elasticity of longevity with respect to health expenditure (henceforth “longevity elasticity”), while the Appendix provides a detailed description of the parameter values and data sources used in the simulations.

### 2.2 Model structure

Based on previous research (Hall and Jones, 2007), we derive from a simple model a macro-efficiency score that indicates whether an economy underspends or overspends on healthcare (see the Appendix for a full derivation). We assume individuals maximize their discounted lifetime utility u(c)⋅DLE(h), as defined by the utility u(c) from annual consumption c multiplied by the individual's discounted life expectancy at birth DLE(h), which can be increased by annual health expenditure h. Note that DLE(h) is a measure (for a precise formulation, see the Appendix) that increases with life expectancy at birth LE(h) and decreases with the rate of time preference at which individuals discount the future (i.e., the extent to which they prefer present day consumption).

Individuals allocate their income to consumption, which raises their utility within each life year, and health investments, which increase life expectancy and allow the spread of consumption utility over additional life years, according to the budget constraint y=c+h. We show in the Appendix that this yields an optimal spending rule, according to which the ratio of health expenditures to consumption expenditures equals the ratio of the elasticity of discounted life expectancy with respect to health expenditure (henceforth “discounted longevity elasticity”), $\frac{DLE^{\prime }\left(h\right)\cdot h}{DLE\left(h\right)},$ and the elasticity of utility with respect to consumption, $\frac{u^{\prime }\left(c\right)\cdot c}{u\left(c\right)}$,

$$\begin{array}{l} \frac{\frac{DLE^{\prime }\left(h\right)\cdot h}{DLE\left(h\right)}}{\frac{u^{\prime }\left(c\right)\cdot c}{u\left(c\right)}}\;=\frac{h}{c} \end{array},$$

where $DLE^{\prime }\left(h\right)$ and $u^{\prime }\left(c\right)$ denote the marginal effects of health care on the discounted life expectancy at birth and on period utility, respectively. The elasticities are defined as the percentage change in discounted longevity and utility for a 1 % increase in health and consumption expenditures, respectively. Thus, they measure the effectiveness of health expenditure versus consumption in raising life-cycle utility.

The optimal spending rule yields the following insights: (i) A richer country would, all else equal, spend more on healthcare relative to consumption. This follows from the fact that a lower elasticity of consumption utility typically characterizes rich countries, reflecting the decreasing effects of additional consumption on utility that follows from the hierarchy of individual needs. For a given discounted longevity elasticity, a rich country is thus willing to devote a greater share of income to healthcare. (ii) It can be shown (see the Appendix) that the discounted longevity elasticity decreases with the level of life expectancy and with the discount rate. Intuitively, countries with lower levels of life expectancy and with more patient populations tend to value more an increase in longevity. (iii) Medical progress that raises the discounted longevity elasticity triggers a reallocation of available income from consumption to healthcare. (iv) Countries that for some reason are less effective in producing health, as measured by a lower discounted longevity elasticity, should, all else equal, spend less on healthcare. While many exogenous factors—such as demographic, social, cultural, and environmental determinants of health—may explain why countries differ in the productivity of their health expenditure, an intricate link exists between production efficiency and macroeconomic efficiency.

Suppose two countries, A and B, share the same level of per capita GDP, but country B runs an inefficient health system and therefore exhibits a lower longevity elasticity. In this case, according to the optimal spending rule, country B should devote fewer resources to healthcare. While redirecting resources away from a relatively inefficient use can be viewed as a “locally” optimal response, it would be misguided in global terms. The appropriate strategy would be to reorganize the health system toward improved efficiency and maintain (or, depending on the starting point, increase) overall health expenditure. Hence, whether “cost containment” reflects warranted efficiency gains or whether it reflects unwarranted spending cuts must be considered very carefully.

In reality, the optimal spending rule may not be met for the following reasons: (i) lacking expert knowledge, individuals typically delegate most of their healthcare choices to physicians who, following their own objectives, may spend non-optimally (Arrow, 1963); (ii) where individuals determine spending levels, lack of information about treatment effectiveness may lead to suboptimal spending decisions; and (iii) where the government and insurance firms determine spending levels, they may follow spending rules that are unrelated to the individual's optimization. For instance, evidence exists that government spending in the United States reflects special interests rather than the median voter's preferences (Gilens and Page, 2014; Page et al., 2013).

To assess the extent of underspending or overspending, we transform the optimal spending rule into a macro-efficiency score **(**Appendix, equation (2)). Underspending on healthcare prevails if the macro-efficiency score is less than 1, and the reverse holds true if the score exceeds 1. By setting the macro-efficiency score equal to 1, we can recalculate optimal health expenditures and, thus, the percentage gap between actual and optimal expenditures.

### 2.3 Output estimates

We calculate the macro-efficiency score for the following countries—Argentina, Australia, Brazil, Canada, China, France, Germany, India, Italy, Japan, the Russian Federation, South Africa, Spain, the United Kingdom, and the United States—to assess how much these countries underspend or overspend relative to their GDP per capita.

Recalling that the utility elasticity with respect to consumption tends to fall with increasing consumption and, by implication, with increasing GDP per capita, determining the longevity elasticity is the only aspect that remains undone. Unfortunately, we lack country-specific data on this elasticity. To obtain an “average” measure, we use data from the World Development Indicators (World Bank, 2021) to regress the log of life expectancy on the log of health expenditures, controlling for the log of per capita income, the share of people aged 65 and older, and the squared terms of the log of per capita income and the share of people above the age of 65. The inclusion of squared terms accounts for nonlinearities in the relationship. For countries with a GDP per capita higher than the global median, we obtain an estimate for the longevity elasticity of 0.051 for the year 2015, implying that a 1 % increase in health expenditure raises life expectancy by 0.051 %. We then use this longevity elasticity to calculate the macro-efficiency score. We also derive the optimal health expenditure for each country. To provide changes across time, we further compute the macro-efficiency score for the year 2010 using the longevity elasticity of 0.061 estimated for the year 2010. The Appendix provides further detail on parameter choices and data sources.

### 2.4 Validity check based on the value of a statistical life

One advantage of our approach is that it allows for computing an implied value of a statistical life (VSL) across countries that we can compare to the estimated value in a country, derived from the literature (Viscusi and Masterman, 2017). Doing so represents an independent validity check of our results on the extent of underspending or overspending on healthcare. See the Appendix for further details on the VSL calculations.

### 2.5 Sensitivity analysis

Furthermore, we conduct a sensitivity analysis using the longevity elasticity of 0.04, as found in a meta-analysis of different estimates (Gallet and Doucouliagos, 2017).

## 3 Results

### 3.1 Macro-efficiency score and the assessment of underspending or overspending on health

Table 1 presents the macro-efficiency scores that indicate whether a country underspends or overspends on healthcare. In 2015, Canada and Japan spent around 10 % of their respective GDPs on healthcare; the United States exhibited considerably higher spending of close to 17 %, whereas China, India, and the Russian Federation spent only around 4 % to 5 % of their GDPs on health. Judging from the macro-efficiency scores, all countries under consideration underinvest in healthcare except the United States. While the United States underspent in 2010, it overspent in 2015, indicating that the current health expenditure share in GDP for the United States is likely to be near the optimal point. On the other side of the range, underinvestment is particularly pronounced in China, India, and the Russian Federation.

**Table 1:** Macro-efficiency score and health expenditure (% of GDP) in 2010 and 2015.

Country	2010		2015	
	Macro-efficiency score	Health expenditure (% of GDP)	Macro-efficiency score	Health expenditure (% of GDP)
Argentina	0.49	9.45	0.54	8.79
Australia	0.43	8.43	0.57	9.31
Brazil	0.42	7.95	0.57	8.87
Canada	0.55	10.68	0.65	10.51
China	0.25	4.21	0.33	4.89
France	0.59	11.24	0.73	11.46
Germany	0.57	11.10	0.68	11.09
India	0.18	3.27	0.24	3.60
Italy	0.46	8.92	0.56	8.99
Japan	0.48	9.16	0.70	10.89
Russian Federation	0.23	4.97	0.30	5.30
South Africa	0.34	7.42	0.48	8.20
Spain	0.47	9.12	0.57	9.11
United Kingdom	0.51	9.99	0.59	9.69
United States	0.85	16.35	1.05	16.84

**Note:** The longevity elasticity is 0.061 and 0.051 for 2010 and 2015, respectively, and is based on our estimates using World Bank data. A macro-efficiency score in excess of 1 indicates overspending, while a score below 1 indicates underspending on healthcare. Health expenditure (% of GDP) is based on World Bank data.

### 3.2 The gap between current and optimal health expenditure in GDP

**Figure 1:**
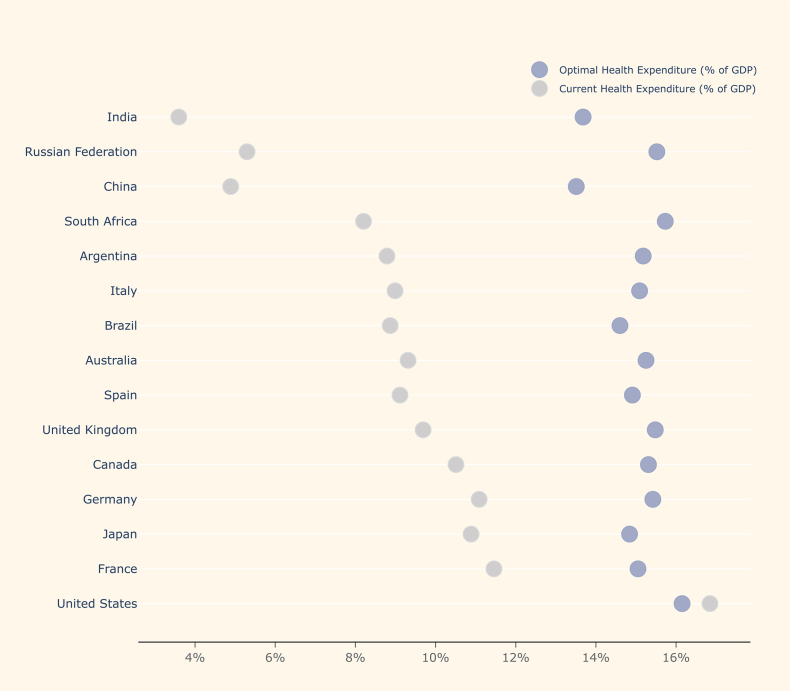
Current and optimal health expenditure shares (% of GDP). **Note**: The countries are sorted based on the percentage gap between the current versus optimal health expenditure share in GDP. The current health expenditure (% of GDP) is based on World Bank data from 2015. The optimal health expenditure (% of GDP) is estimated from our framework using the longevity elasticity of 0.051.

Figure 1 shows the gap between current and optimal health expenditure in GDP for each country in 2015. The countries are ranked by the gap between current and optimal health expenditure in GDP, which is largest in India (67.4 %), followed by the Russian Federation (65.9 %), China (63.8 %), and South Africa (47.9 %). In contrast, the United States overspent by 4.3 %.

### 3.3 Validity check based on the value of a statistical life

Table 2 calculates for our model the VSL as a measure of how much individuals would be willing to pay for an instantaneous reduction in mortality (Hall and Jones, 2007; Murphy and Topel, 2006) and compares it with the micro-econometric estimates of Viscusi and Masterman (2017). Although the VSL values are derived by very different methods, they are of the same order of magnitude, underscoring the validity of our computations.

**Table 2:** Comparison between VSL estimates (in million USD).

Country	VSL (model)		VSL (estimated)	
	2010	2015		
Argentina	3.99	4.20	2.14	
Australia	7.27	7.96	10.34	
Brazil	2.21	2.41	1.70	
Canada	7.36	7.99	8.18	
China	0.66	1.11	1.36	
France	6.65	6.71	6.98	
Germany	7.40	7.78	7.90	
India	0.47	0.68	0.28	
Italy	7.48	7.04	5.65	
Japan	6.17	6.40	6.68	
Russian Federation	3.09	3.44	1.97	
South Africa	1.62	1.75	1.05	
Spain	6.13	6.21	4.91	
United Kingdom	7.77	8.39	7.47	
United States	10.85	11.68	9.63	

**Note:** The longevity elasticity is 0.061 and 0.051 for 2010 and 2015, respectively, and is based on our estimates using World Bank data. The VSL (model) is calculated within our framework, while the VSL (estimated) is from Viscusi and Masterman (2017).

### 3.4 Sensitivity analysis

We then conducted a sensitivity analysis using the lower longevity elasticity estimate of 0.04, taken from a recent meta-analysis (Gallet and Doucouliagos, 2017). The results remain robust, with India, the Russian Federation, and China having the lowest and the United States having the highest macro-efficiency scores (see Table 3). All countries except for the United States underspend on health.

**Table 3:** Macro-efficiency score and health expenditure (% of GDP) in 2010 and 2015 using a longevity elasticity estimate of 0.04 (Gallet and Doucouliagos, 2017).

Country	2010		2015	
	Macro-efficiency score	Health expenditure (% of GDP)	Macro-efficiency score	Health expenditure (% of GDP)
Argentina	0.74	9.45	0.69	8.79
Australia	0.66	8.43	0.73	9.31
Brazil	0.64	7.95	0.73	8.87
Canada	0.85	10.68	0.83	10.51
China	0.38	4.21	0.42	4.89
France	0.91	11.24	0.93	11.46
Germany	0.87	11.10	0.88	11.09
India	0.28	3.27	0.30	3.60
Italy	0.70	8.92	0.71	8.99
Japan	0.74	9.16	0.90	10.89
Russian Federation	0.36	4.97	0.39	5.30
South Africa	0.53	7.42	0.61	8.20
Spain	0.73	9.12	0.73	9.11
United Kingdom	0.78	9.99	0.75	9.69
United States	1.31	16.35	1.35	16.84

**Note:** The longevity elasticity of 0.04 is estimated within a meta-regression consisting of 65 studies completed over the 1969–2014 period (Gallet and Doucouliagos, 2017). A macro-efficiency score in excess of 1 indicates overspending, while a score below 1 indicates underspending on healthcare. Health expenditure (% of GDP) is based on World Bank data.

## 4 Discussion

### 4.1 Underspending on healthcare

This study, for the first time, estimates the macro-level efficiency of health expenditure for 15 countries. We find that from a macroeconomic perspective, almost all countries included in our analysis would benefit from increasing their health expenditure. Despite their relatively high health expenditure shares, countries such as Argentina, Australia, Canada, Italy, Japan, and the United Kingdom would benefit from further expansion, as the high GDP levels in these countries translate into large VSLs. Underinvestment is particularly pronounced in China, India, and the Russian Federation due to the low share of health expenditures in GDP, which might be due to these countries following other priorities in spending, such as on infrastructure in the case of China (Chen et al., 2020).

In general, the optimal shares of health expenditure in GDP are similar across countries, ranging between 14 % in China and India to 16 % in the United States. Although the lower GDP and associated VSL in China and India imply a lower optimal share of health expenditures in GDP, our macro-efficiency score indicates that the current spending shares are so low that these two countries would particularly benefit from spending increases. The situation is somewhat different for the Russian Federation and for South Africa, with both countries exhibiting optimal shares of health expenditures in GDP that are comparable to or even exceed those of countries with higher GDPs per capita. Here the reason lies in the low levels of life expectancy, which tend to support a higher optimal share of health expenditures in GDP. Generally, given the longevity elasticity, the optimal health expenditure share in GDP is explained by both GDP per capita (particularly low in China and India; particularly high in the United States) pointing at a higher spending share and life expectancy (particularly low in the Russian Federation, South Africa, and India; particularly high in Japan) pointing at a lower spending share.

Overall, the United States is the only country exhibiting overspending, particularly in 2015. The explanation for this is rooted in the relatively high share of health expenditure in GDP. To decidedly answer the question raised by Garber and Skinner (2008), “Is American healthcare uniquely inefficient?” we would, however, require more precise estimates of the longevity elasticity for the United States.

Our study emphasizes that the major and urgent issue in many countries is underinvestment in health at the macroeconomic level, rather than containing costs at the microeconomic level. Countries should further expand their healthcare sectors by increasing investment and promoting innovation of high-quality and effective care that can increase longevity. While such an expansion should be executed in a way that is micro-efficient, our analysis shows that an exclusive focus on expenditure containment is inappropriate. Health expenditure should not be viewed simply as a burden; rather, it is a reflection of countries’ modernization and respect for their citizens, whose wellbeing depends decisively on living long and healthy lives.

### 4.2 Valuing health at the macroeconomic level

Our result that most countries included in our analysis underspend on healthcare is based on a relatively narrow macro-efficiency criterion relating to income and the effectiveness of healthcare in raising life expectancy. We would expect an even stronger case for investments in health and healthcare within a broader macroeconomic framework.

First, investments in health and healthcare can drive economic growth by improving population health. Healthier populations tend to have higher labor force participation rates, greater productivity, and longer working lifespans (Bloom and Canning, 2000; Bloom et al., 2019a; Bloom et al., 2020; Bloom et al., 2019b; Bloom et al., 2018; Bloom et al., 2019c; Chen and Bloom, 2019; Chen et al., 2018; Chen et al., 2019a, b; Weil, 2007). With more of the population in employment and a higher production potential for each worker, a country with a healthier population can achieve higher per capita output. Longer life expectancy also incentivizes savings, education, and investment in research and development (R&D), which in turn contribute to economic growth (Ben-Porath, 1967; Bloom and Canning, 2000; Bloom et al., 2003; Cervellati & Sunde, 2005, 2013, 2013; Gehringer and Prettner, 2019; Prettner and Trimborn, 2017). These dynamic effects are, at best, incompletely reflected in the individual's willingness to pay for healthcare and, thus, tend to imply that our macro-efficiency score is prone to underestimate the spending level required to attain macroeconomic efficiency in an economic growth context.

Second, medical and healthcare services can also drive economic growth through technological innovation. Studies show that, as the size and value of the healthcare market increases, the extent of R&D activity on pharmaceuticals and advanced medical technology and their diffusion expands (Acemoglu and Linn, 2004; Clemens, 2013; Finkelstein, 2004, 2007, 2007; Frankovic and Kuhn, 2018; Frankovic et al., 2020). Large welfare gains can also be achieved even if many of the ensuing innovations are not cost-effective (Böhm et al., 2021; Chandra and Skinner, 2012; Fonseca et al., 2020; Frankovic and Kuhn, 2018; Frankovic et al., 2020), which follows as a corollary to our finding that income growth should increasingly translate into health expenditure. Notably, this case is weakened to the extent that medical spending is subject to decreasing returns, leading to “flat-of-the-curve” medicine. Medical progress is then valuable in shifting the whole health production function upward, affording an increase in medical productivity throughout. Jones (2016) shows that under plausible assumptions about preferences, this is true even if medical R&D crowds out innovations aimed at conventional productivity growth.

Third, investments in health and healthcare can also improve the quality and resilience of health systems, which can ensure social stability, especially in an emergency. A modern health system with a certain excess capacity in terms of equipment, beds, and staff can be viewed as a form of insurance against large-sized medical incidents, such as accidents involving mass casualties, natural or manmade disasters, and epidemic outbreaks of infectious diseases (Attema et al., 2010; Lakdawalla et al., 2017; Megiddo et al., 2019; Philipson and Zanjani, 2014; Zweifel et al., 2009; Chen et al., 2020a, 2020b). In today's interconnected world, emerging infectious diseases with pandemic potential can cost millions of lives, cause economic upheaval, and disrupt travel and trade (Acemoglu et al., 2020; Eichenbaum et al., 2020; Glover et al., 2020; International Monetary Fund; International Monetary Fund, 2020; Krueger et al., 2020; U.S. Department of Health and Human Services, 2010; Chen et al., 2020, Chen et al., 2021a, Chen et al., 2021b). Developing modern health systems, advanced medical and healthcare technology, and sufficient capacity in terms of facilities, equipment, and trained staff are crucial to ensure not only health system stability, but social and economic stability as well.

### 4.3 Strengths

Against a large body of evidence on the productive efficiency of healthcare in various settings, this study is the first to provide an intuitive yet rigorous test for the macroeconomic efficiency of health expenditure. One strength is that we derive the macro-efficiency score from economic theory. A second strength is that the macro-efficiency score can be calculated based on relatively easily accessible data, although the test can, in principle, be extended to more complex settings and/or detailed data. Our approach allows for a straightforward assessment and ranking of the extent to which countries underspend or overspend on healthcare.

### 4.4 Limitations

Our analysis has several caveats. First and most important, our test only allows us to assess the deviation from a macro-efficient spending level that is conditional on average production efficiency, as measured by the estimated longevity elasticity. Thus, while our conservative use of a lower estimate of the longevity elasticity than the one we estimate provides a robust assessment of the direction of macro-inefficiency for the countries under consideration, we cannot disentangle macro-inefficiency from micro-inefficiency (i.e., production inefficiency) at the country level. This implies that the measured macro-inefficiency (i.e., the deviation from 1) may to some extent be upward or downward biased.

Second, our results are preconditioned on our use of life expectancy at birth as the outcome criterion. While this is consistent with the underlying framework in Hall and Jones (2007), it fails to incorporate explicitly quality-of-life dimensions of healthcare. That said, life expectancy is a plausible (long-run) proxy for health in itself (Vaupel, 2010), while, in economic terms, quality of life is a close complement to length of life in generating utility (Hall and Jones, 2007; Murphy and Topel, 2006). Assuming that countries choose a mix of healthcare that balances length of life and quality of life, one can then interpret life expectancy as a sufficient statistic for quality-related aspects of health as well (Chandra and Skinner, 2012). On these grounds, we argue for the robustness of our results when it comes to assessing the macro-efficiency of healthcare spending.

Third, our test is based on the assumptions that (i) the social benefits of health expenditure equal the private benefit of greater longevity (including quality of life for the sake of the argument), while (ii) the opportunity cost of health expenditure is appropriately measured by the marginal utility of foregone consumption. Both (i) and (ii) require qualification, as (i) some interventions such as vaccinations not only improve an individual's private health but also generate positive externalities by improving population health, and (ii) the marginal utility of foregone consumption appropriately measures the opportunity cost of spending only when assuming that other investments chosen by the individual follow a similar optimality rule as that outlined in the optimal spending rule (Appendix, equation (1))**.** In particular, this is important in the context of spending on education. Extensive evidence exists on the complementarity of health and education as components of human capital, implying that underinvestment in one domain is associated with underinvestment in the other domain as well (Bleakley, 2007, 2010, 2010; Cervellati & Sunde, 2013, 2015, 2015; Field et al., 2009; Hansen and Strulik, 2017; Jayachandran and Lleras-Muney, 2009; Lleras-Muney, 2005; Lucas, 2010; Miguel and Kremer, 2004). This suggests that our findings are on the conservative side. We provide further analysis and discussion in the Appendix.

Fourth, we cannot easily extend our analysis to low-income countries. When running the regression for the countries below the median world income, the longevity elasticity is insignificant, mainly because mortality is determined by many aspects that are not closely related to health expenditures in these countries, such as hunger and a lack of clean drinking water. Thus, we only include middle- and high-income countries in this study.

We conclude by noting that all of these caveats predominantly relate to measurement issues, which the use of more elaborate indicators and/or additional data can address. They do not, however, compromise the general design and applicability of our test for macro-efficiency.

## 5 Conclusion

While the understanding of efficiency in health spending is robust at the micro- and meso-levels, the efficiency of health expenditure at the macroeconomic level is less studied and understood. We developed a score to assess the macro-efficiency of health expenditure and found that Argentina, Australia, Brazil, Canada, China, France, Germany, India, Italy, Japan, the Russian Federation, South Africa, Spain, and the United Kingdom all underspend on healthcare and would benefit from devoting more resources to their healthcare sectors. The United States is the only country exhibiting overspending. Our study emphasizes that the major and urgent issue in many countries is underinvestment in health at the macroeconomic level, rather than containing costs at the microeconomic level. Health, healthcare, and medical science are essential to a citizenry's welfare and are important for social stability. Health expenditure should not be viewed simply as a burden; rather, it is a reflection of a country's modernization and respect for its citizens, whose wellbeing depends decisively on living long and healthy lives. Looking forward, a high-quality, responsive, and resilient health system and an efficient and innovative mechanism to promote R&D in public health and medical sciences are urgently needed more than ever.
