# Supplementary material for: Macro-level efficiency of health expenditure: Estimates for 15 major economies
Source: Soc Sci Med. 2021 Oct;287:114270. doi: 10.1016/j.socscimed.2021.114270 (PMC8412416; doi:10.1016/j.socscimed.2021.114270)
Supplement: Multimedia component 1 [file mmc1.docx]

**Appendix: Macro-level efficiency of health expenditure: estimates for 15 major economies**

This Appendix provides additional details related to our study, including the mathematical formulation of our model and detailed data sources.

**A. Mathematical Formulation**

Based on Hall and Jones (2007)(Hall & Jones, 2007), we develop a simple macroeconomic test of whether an economy under- or overinvests in healthcare. To this end, suppose individuals maximize their discounted lifetime utility,

$$u\left( c \right)\cdot DLE\left( h \right),$$

i.e., the instantaneous utility $u\left( c \right)$from annual consumption, $c$, multiplied by the discounted life expectancy at birth (henceforth discounted longevity) $DLE\left( h \right)$, depending on the level of annual health expenditure, $h$. Denoting annual income of the representative individual by $y$ and abstracting from saving, the budget constraint reads

$y=c+h$.

Solving the utility maximization problem leads to the following optimality condition for the ratio of consumption expenditures to health expenditures:

$$\begin{aligned} \frac{\frac{DLE^{'}\left( h \right)\cdot h}{DLE\left( h \right)}}{\frac{u^{'}\left( c \right)\cdot c}{u\left( c \right)}} =\frac{h}{c} .\#\left( 1 \right) \end{aligned}$$

In this expression, the left-hand side is given by the ratio of the elasticity of discounted longevity with respect to health expenditure and the elasticity of utility with respect to consumption. The former measures the effectiveness of healthcare in expanding life, and the latter measures the contribution of consumption to wellbeing. Note that both elasticities are measured in relation to current levels of health expenditure and consumption, respectively. According to **equation (1),** utility is maximized by equalizing the ratio of health expenditures to consumption expenditures with the ratio of their corresponding elasticities. For reference, note that the health-to-consumption expenditure ratio, $h/c$, can also be written as an increasing function of the health expenditure share in GDP, $h/y$, where $\frac{h}{c}=\frac{h/y}{1-\left( h/y \right)}.$

To assess the extent of under- or over-spending, we reformulate the **optimality condition (1)** to get

$$\begin{aligned} \frac{\frac{h}{c}}{\frac{DLE^{'}\left( h \right)\cdot h}{DLE\left( h \right)} \cdot\frac{u\left( c \right)}{u^{'}\left( c \right)\cdot c}}⋚1,\#\left( 2 \right) \end{aligned}$$

where we define the left-hand side as our macro-efficiency score. If this score is smaller than one, underspending on healthcare prevails. The reverse is true if the macro-efficiency score is greater than one. Note that, for given data on consumption and a given elasticity of longevity, we can use **equation (2)** to calculate the optimal health expenditures $h^{*}$ at which the macro-efficiency score equals unity.

**B. Data Sources and Parameters**

To perform the test for 15 major economies, we rely on World Development Indicators (2021) data on life expectancy, consumption expenditures as a share of GDP, health expenditures as a share of GDP, and GDP itself (World Bank, 2021). As a specification for instantaneous utility, we apply a standard isoelastic utility function:

$u\left( c \right)= \frac{c^{1-\theta}-1}{1-\theta}$,

where $\theta\in\left( 0,\infty\right)$ is the inverse of the elasticity of intertemporal substitution, i.e., a measure of how willing households are to sacrifice consumption today in exchange for consumption tomorrow. The parameter determines the curvature of the utility function in that a higher $\theta$ implies a more concave function and households that are less willing to sacrifice consumption today for consumption tomorrow. We assume $\theta=1.01$, which is well in line with empirical evidence (Chetty, 2006). In addition, and in extension to the simple model presented in Section A, we consider that individuals discount future utility. Specifically, we consider a model where the representative individual (i) faces a mortality rate $\mu\left( h \right)$ within each period, implying an (undiscounted) life expectancy $LE\left( h \right)={\mu\left( h \right)}^{-1}$, and (ii) discounts the future at the rate $\rho\geq0$. In this case, the discounted life expectancy is given by $DLE\left( h \right)=\left[ \mu\left( h \right)+\rho\right]^{-1}$, and life-cycle utility can be written as $u\left( c \right)\left[ \mu\left( h \right)+\rho\right]^{-1}$ (Kuhn & Prettner, 2016). Noting that $\frac{LE\left( h \right)^{'}h}{LE\left( h \right)}=\frac{-\mu\left( h \right)^{'}h}{\mu\left( h \right)}$ we can write the elasticity of discounted longevity with respect to health expenditure

$$\frac{{DLE\left( h \right)}^{'}h}{DLE\left( h \right)}=\frac{-\mu\left( h \right)^{'}h}{\mu\left( h \right)+\rho}= \frac{-\mu\left( h \right)}{\mu\left( h \right)+\rho}\frac{\mu\left( h \right)^{'}h}{\mu\left( h \right)}= \frac{DLE\left( h \right)}{LE\left( h \right)}\frac{LE\left( h \right)^{'}h}{LE\left( h \right)}$$

as the product of the elasticity of (undiscounted) longevity with respect to health expenditure, $\frac{LE\left( h \right)^{'}h}{LE\left( h \right)}$, and a weight $\frac{DLE\left( h \right)}{LE\left( h \right)}\leq1$, which is a decreasing function of life expectancy $LE\left( h \right)$ and the discount rate. We assume a discount rate of $\rho=0.025$, which we subsume into the discounted life expectancy term. The main text provides details of our estimation of the elasticity of longevity with respect to health expenditure $\frac{LE\left( h \right)^{'}h}{LE\left( h \right)}$.

**C. Value of Statistical Life (VSL) Calculations**

In our framework, the VSL is given by ${u\left( c \right)}/{u'\left( c \right)}\cdot DLE\left( h \right),$ i.e., as the product of the monetary value of a life year, ${u\left( c \right)}/{u'\left( c \right)},$ and discounted life expectancy. Note that the division of utility (measured in abstract “utils”) by marginal utility (measured in “utils per dollar”) leads to a monetary (dollar) value that allows for a direct comparison with the estimated dollar value in the literature.

**D. Relation between Healthcare and Education**

The following describes a possible argument against assessing the macroeconomic efficiency of healthcare spending based on the spending rule in the optimality condition (**equation (1)**). The marginal utility of consumption may not be the appropriate measure of opportunity cost, as it does not measure the returns to education or to investments in infrastructure or environmental protection unless these investments are chosen according to a similar rule. While this places a caveat on our analysis, one can draw on the extensive evidence on the complementarity among health, longevity, and education to argue that wherever underspending on healthcare is large, underspending on education may also be large (Bleakley, 2007, 2010; Cervellati & Sunde, 2013, 2015; Field et al., 2009; Hansen & Strulik, 2017; Jayachandran & Lleras-Muney, 2009; Lleras-Muney, 2005; Lucas, 2010; Miguel & Kremer, 2004). In the following, we will formalize this notion by drawing on an extension of our model to incorporate an up-front investment in education.

To this end, assume that during early adulthood, call this period 0, individuals face an option to make a once-and-for-all investment in education, $e$, which raises their income $y\left( e \right)$ during their subsequent working lives. Thus, consider individuals to maximize their discounted lifetime utility,

$$u_{0}\left( c_{0} \right)+u\left( c \right)\cdot DLE\left( h \right),$$

where $u\left( c \right)\cdot DLE\left( h \right)$ is the discounted stream of utility, depending on annual consumption $c$ and health expenditure, $h$, during working life, and where $u_{0}\left( c_{0} \right)$ is the utility from consumption during early adulthood, $c_{0}$. For ease of exposition, assume that individuals are unable to shift income across the life-cycle and, thus, face the set of budget constraints

$$y_{0}-e=c_{0}$$

during early adulthood, where $y_{0}$ is (potential) income, and

$$y\left( e \right)=c+h$$

during each year of their working lives. It is easy to show that the model carries over to a setting in which the individuals face a life-cycle budget constraint.

Substituting from the budget constraints into the utility function gives $u_{0}\left( y_{0}-e \right)+u\left( y\left( e \right)-h \right)\cdot LE\left( h \right),$ and maximizing this with respect to $h$ and $e$ gives us the two first-order conditions

$$\begin{aligned} -u^{'}\cdot DLE+u\cdot DLE'=0,\#\left( 3 \right) \end{aligned}$$

$$\begin{aligned} {-u'}_{0}+u^{'}y^{'}\cdot DLE=0.\#\left( 4 \right) \end{aligned}$$

Under the common-place assumptions of positive and decreasing marginal utility ($u^{'},{u'}_{0}>0,{u^{'}}^{'},{u^{''}}_{0}<0$) and positive and (weakly) decreasing returns to health investments ($DLE^{'}>0,DLE''\leq0$) and educational investments ($y^{'}>0,y^{''}\leq0$), one can show that a set of upward-sloping “best-responses” $\hat{h}\left( e \right)$ and $\hat{e}\left( h \right)$ exist, which (under common-place assumptions) form a unique and stable optimum at their point of intersection $h^{*}=\hat{h}\left( e^{*} \right)$ and $e^{*}=\hat{e}\left( h^{*} \right)$.

The joint complementarity between investments into health and education, i.e. $\hat{h}^{'}>0$ and $\hat{e}^{'}>0$, can be gleaned intuitively from the two **first-order conditions (3)** and **(4)**. Considering the **optimal investment rule (3)** for health, we note that the return on investment in longevity expansion, as measured by period utility, $u$, increases with $y\left( e \right)$ and, thus, with $e$. At the same time, the marginal utility cost of health investments, $u'$, decreases with $y\left( e \right)$. Hence, the additional income afforded by higher education increases both the return on health investments and their affordability. Considering the **optimal investment rule (4)** for education, we note that the return on education increases with health investments, $h,$ through two channels: (i) the well-known Ben-Porath effect, whereby a (health-driven) increase in longevity $LE\left( h \right)$ increases the return on education (Cervellati & Sunde, 2013; Hansen & Strulik, 2017; Jayachandran & Lleras-Muney, 2009) and, (ii) an increase in the marginal utility of income, $u^{'},$ during working lives, where additional earnings are valued for the purpose of financing (old-age) health care needs.

Taken together, the joint complementarity of investments in health and education suggests that an under-investment in health, i.e. $h<h^{*}$ $,$ triggers an under-investment in education relative to the (unconditional) optimum, $e^{*},$ even when triggering a (conditionally) optimal response, $\hat{e}\left( h \right)$, i.e. we have $e=\hat{e}\left( h \right)<\hat{e}\left( h^{*} \right)=e^{*}.$ In terms of our analysis, this implies that the identified tendency towards under-investment in health is indicative of an equal tendency towards under-investment in education (relative to the unconditional optimum). Considering the reverse channel, we find that this is suggestive of a reinforcement of under-investment in health, where $\hat{h}\left( e \right)<\hat{h}\left( e^{*} \right)=h^{*}$, which is strengthening our finding. Indeed, quite possibly our findings of underinvestment in health care are indicative of economies being trapped in an equilibrium with under-investment in both dimensions. Whether or not this is borne out by the data is an issue we relegate to future research.

**References**

Bleakley, H. (2007). Disease and development: evidence from hookworm eradication in the American South. *The Quarterly Journal of Economics,* 122, 73-117.

Bleakley, H. (2010). Malaria eradication in the Americas: A retrospective analysis of childhood exposure. *American Economic Journal: Applied Economics,* 2, 1-45.

Cervellati, M., & Sunde, U. (2013). Life expectancy, schooling, and lifetime labor supply: theory and evidence revisited. *Econometrica,* 81, 2055-2086.

Cervellati, M., & Sunde, U. (2015). The effect of life expectancy on education and population dynamics. *Empirical Economics,* 48, 1445-1478.

Chetty, R. (2006). A new method of estimating risk aversion. *American Economic Review,* 96, 1821-1834.

Field, E., Robles, O., & Torero, M. (2009). Iodine deficiency and schooling attainment in Tanzania. *American Economic Journal: Applied Economics,* 1, 140-169.

Hall, R.E., & Jones, C.I. (2007). The value of life and the rise in health spending. *The Quarterly Journal of Economics,* 122, 39-72.

Hansen, C.W., & Strulik, H. (2017). Life expectancy and education: evidence from the cardiovascular revolution. *Journal of Economic Growth,* 22, 421-450.

Jayachandran, S., & Lleras-Muney, A. (2009). Life expectancy and human capital investments: Evidence from maternal mortality declines. *The Quarterly Journal of Economics,* 124, 349-397.

Kuhn, M., & Prettner, K. (2016). Growth and welfare effects of health care in knowledge-based economies. *Journal of Health Economics,* 46, 100–119.

Lleras-Muney, A. (2005). The relationship between education and adult mortality in the United States. *The Review of economic studies,* 72, 189-221.

Lucas, A.M. (2010). Malaria eradication and educational attainment: evidence from Paraguay and Sri Lanka. *American Economic Journal: Applied Economics,* 2, 46-71.

Miguel, E., & Kremer, M. (2004). Worms: identifying impacts on education and health in the presence of treatment externalities. *Econometrica,* 72, 159-217.

World Bank. (2021). World Development Indicators.
